# Novel *PAX6* mutation reported in an aniridia patient

**DOI:** 10.1038/hgv.2017.53

**Published:** 2017-12-07

**Authors:** Andrew Winegarner, Yoshinori Oie, Satoshi Kawasaki, Nozomi Nishida, Kohji Nishida

**Affiliations:** 1 Department of Ophthalmology, Osaka University Graduate School of Medicine, Osaka, Japan; 2 Department of Ophthalmology, Kansas University Medical Center, School of Medicine, Kansas City, KS, USA

## Abstract

An aniridia patient was found to have a novel *PAX6* mutation. A genetic duplication within *PAX6*, which caused a frameshift mutation, ultimately created a nonsense stop codon and premature truncation of the protein. Consequently, the patient presented with a clouded cornea as a result of partial limbal stem cell deficiency, foveal hypoplasia, nystagmus and a pale, cupped optic disc caused by glaucoma.

Aniridia primarily occurs due to mutations of *PAX6* on band p13 of chromosome 11. Furthermore, aniridia is a panocular syndrome that affects the cornea, anterior chamber, iris, lens, retina, macula and optic nerve.^1^ Approximately 66% of aniridia cases are familial, while 33% are sporadic.^2^ However, 90% of aniridia cases are related to *PAX6* gene mutations, regardless of familial or sporadic status.^3^ Single allele mutations seem to be haploinsufficient for some cells and tissue. Dual allele mutations appear to be fatal since the absence of *PAX6* gene expression may cause defects in brain development.^4^ Clinically, aniridia patients often present with iris hypoplasia, cataracts, glaucoma, corneal opacity, foveal hypoplasia, and nystagmus.^3^ In the present study, we demonstrate a novel *PAX6* mutation in an aniridia patient.

A 64-year-old diabetic Japanese male presented with significant bilateral iris hypoplasia and corneal opacity as a result of partial limbal stem cell deficiency and corneal stromal scarring (Figure 1a and b). His visual acuity was 20/667 in the right eye and 20/2000 in the left eye. A fundus examination revealed a pale optic disc with large cupping (Figure 1c). Additionally, macular hypoplasia was observed via optical coherence tomography (Figure 1d), along with nystagmus. Goldman visual field examination indicated severe visual field loss in the left eye, suggesting glaucoma in accordance with the pale, large cupping optic disc previously described (Figure 1e). Thereafter, we suspected aniridia and conducted a genotype analysis. A penetrating keratoplasty was performed on the right eye to treat the worsening corneal opacity (Figure 1f).

All experimental procedures were approved by the Institutional Review Board of Human Studies at Osaka University. Analysis and research were done in accordance with the tenets of the Declaration of Helsinki for research involving human subjects.

Genomic DNA was extracted from the patient’s buccal epithelial cells using a commercially available kit. All of the *PAX6* exons were amplified by PCR in a 10 μl solution. The PCR products were then treated using ExoSAP-IT (GE Healthcare UK, Buckinghamshire, UK). The treated PCR products were sequenced (BigDye 3.1; ThermoFisher Scientific Inc., Foster City, CA, USA), purified using a commercially available kit (BigDye XTerminator Purification Kit, Applied Biosystems), electrophoresed on an automated sequencer (3130x1 Genetic Analyzer; Applied Biosystems) and finally analyzed with sequence alignment software (Variant Reporter Version 1.0; Applied Biosystems 3730 DNA Analyzer). The conditions for all thermal cycles involved were 30 cycles of three-temperature thermal cycles including denaturation at 94 °C for 30 s, annealing at 55 °C for 30 s, and extension at 72 °C for 30 s.

DNA sequencing of the *PAX6* gene coding and flanking regions revealed a *PAX6* deficiency with a novel mutation. Specifically, this frameshift mutation occurred in exon 7, with a duplication of 4 nucleotides (TTGG) at position 483-486 (c.483_486dupTTGG). This duplication, in turn, caused a frameshift type amino acid sequence change that transitioned tyrosine to leucine at amino acid residue 163 and produced a nonsense stop codon 38 amino acids after the transition (p.Tyr163Leufs*38; Figure 2a–c). This alteration may have caused either a loss of function of the *PAX6* gene in one allele of the patient or decreased mRNA expression via a nonsense-mediated mRNA decay (NMD) mechanism. In both situations, these alterations could lead to a 50% reduction of PAX6 protein expression, resulting in haploinsufficiency, which agrees with the patient’s clinical findings.

Aniridia is most commonly correlated with *PAX6* mutations;^1,5^ however, the number of catalogued mutations is continuously growing. The mutation discovered in this report had not been previously recorded in the Leiden Open Variation Database (LOVD; http://www.lovd.nl/3.0/home). To date, there has not been substantive evidence to support concrete genotype-phenotype correlations.^5,6^ However, as more and more mutations are catalogued, trends may begin to emerge in larger data sets. Other factors may contribute to the phenotype besides the type of mutation involved in *PAX6* haploinsufficient aniridia patients.^7–9^ Thus, this may complicate endeavors to establish more definitive correlations between the mutations and clinical phenotypes.

Beyond haploinsufficiency causing a 50% reduction in *PAX6*, altered *PAX6* may bind to DNA sequences intended for the wild-type PAX6 transcription factor. Thus, inhibition of the remaining wild-type PAX6 transcription factor function would yield dominant negative activity, causing a >50% reduction in *PAX6* function. The binding strength and subsequent inhibition of the wild-type PAX6 transcription factor likely depends on the mutation location and type. This might explain the *PAX6* mutations’ varied severity in clinical phenotypes despite being a heterozygous mutation.^10–12^

Another potential explanation for varying degrees of clinical manifestation among patients with the same mutation may be variations in NMD. NMD degrades premature termination codon-containing transcripts,^13^ such as those commonly seen in aniridia patients. Typically, NMD recognizes premature stop codons at the boundaries between exons and introns; however, NMD generally does not check the last 50 base pairs of the penultimate exon to the end of the gene. Thus, in *PAX6*, at 50 base pairs from the end of exon 12 (base 1496 and onwards), NMD does not check for premature stop codons.^14^ As such, any mutations from the end of exon 12 to exon 13 would theoretically escape NMD recognition and produce the truncated PAX6 protein. This truncated PAX6 protein would bind DNA sequences intended for wild-type PAX6, causing a dominant negative effect. However, databases of recorded *PAX6* mutations do not correlate with these regions despite the fact it should statistically identifiable. A previous report hypothesized that the reason for this is a premature stop codon in this region escapes NMD. This would cause a strong and ultimately fatal dominant negative effect that would not be represented in the mutations catalogued thus far.^14^

More work remains to be done regarding the exploration of genotype-phenotype correlations. However, a crucial initial step is to continue cataloguing novel mutations and reporting their respective clinical scenarios, as accomplished in this report.

## Additional information

**Publisher's note:** Springer Nature remains neutral with regard to jurisdictional claims in published maps and institutional affiliations.

## Figures and Tables

**Figure 1:**
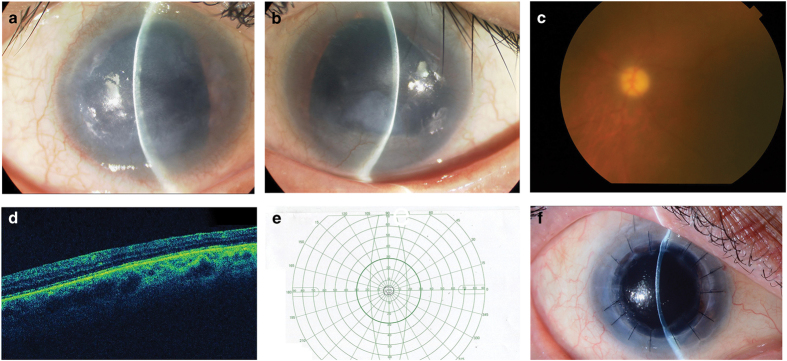
(**a**) Opacified right cornea with iris hypoplasia and corneal stromal scarring due to limbal stem cell deficiency. (**b**) Opacified left cornea with iris hypoplasia and corneal stromal scarring from limbal stem cell deficiency. (**c**) Fundus examination of the left eye reveals a large, pale optic disc with cupping, indicative of glaucoma. (**d**) Optical coherence tomography indicates macular hypoplasia. (**e**) Goldman visual field examination shows significant visual field loss in the left eye, further indicating glaucoma. (**f**) Penetrating keratoplasty was successfully performed on the right eye to treat worsening corneal opacity.

**Figure 2:**
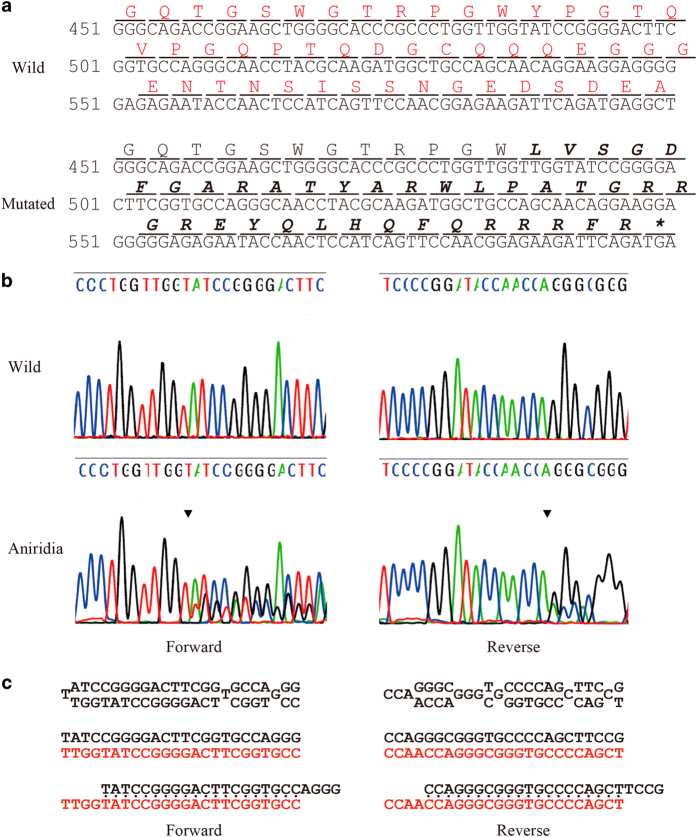
Results of sequencing analysis for the aniridia patient are shown. (**a**) Nucleotide and amino acid sequences of the wild-type (upper) and mutated (lower) *PAX6* gene near the identified p.Tyr163Leufs*38 mutation. The 4-bp duplication of the c.483_486dupTTGG mutation is indicated in bold type in the wild-type sequence. The altered amino acid sequence downstream of the 4-bp duplication is indicated in bold italics in the mutated sequence. An asterisk (*) indicates an ochre (TGA) stop codon. (**b**) Results of sequencing analysis for exon 7 of the *PAX6* gene in a normal volunteer (upper) and the aniridia patient (lower) from the forward (left) and reverse (right) directions. Arrowheads indicate the breakpoint of the c.483_486dupTTGG mutation. (**c**) The mixed base sequence (upper) downstream of the presumed break-points was subtracted (middle) from the reference sequence (black type) to extract the mutated sequence (red type) in both directions (left: forward, right: reverse). Note that the mutated sequence is fully matched to the reference sequence from four bases downstream of the breakpoints (lower), indicating that the mutated sequence is duplicated with four bases (i.e., TTGG).
